# The “secrets” of English private tutoring in China: Chinese students’ experiences of study abroad test preparation in cram schools

**DOI:** 10.3389/fpsyg.2023.1120768

**Published:** 2023-02-15

**Authors:** Cong Zhang, Yi Zhang, Yun Gao

**Affiliations:** School of Foreign Languages and Literature, Shandong University, Jinan, China

**Keywords:** English private tutoring, Chinese students, perceptions, study abroad, shadow education, test preparation

## Abstract

English private tutoring, a.k.a. “shadow education” has been an important way that international students rely on for overseas test preparation. Despite the plethora of research on private tutoring in various countries and regions, scarce research focuses on the type of EPT that prepares students for overseas tests. Therefore, this study investigated the experience and perceptions of EPT in preparing for overseas writing tests of 187 Chinese students through retrospective interviews and questionnaires. The present study investigated Chinese students’ experience in and perceptions of EPT for study abroad writing test preparation. The results showed that most students received EPT in writing in various ways at cram schools and they devoted themselves to EPT in cram schools intensively. They favored EPT in cram schools mainly because the test-taking strategies taught there could help them obtain a higher grade on the writing section in overseas tests. With respect to writing teaching in cram schools, the most frequent teaching activities included teaching test-taking strategies and providing writing templates. Although most students acknowledged the usefulness of EPT in writing in terms of preparing themselves for the writing test, it was not necessarily the case for improving their general writing abilities. The students believed that the writing instruction was test-oriented and had a ceiling effect in improving their general writing abilities. However, with enough long time in EPT, the cramming nature of cram schools can be less obvious.

## 1. Introduction

Private supplementary tutoring has become popular among students to supplement school learning (Yung, 2015). With a quick definition being “tutoring in academic subjects which is provided by the tutors for financial gain and which is additional to the provision by mainstream schooling” (Bray and Kwok, 2003, p. 612), private tutoring has become a widespread phenomenon all over the world. Students participate in private tutoring for various purposes, either remedial or enhancement. It is a crucial but unacknowledged factor in student performance, which can take different forms: one-to-one tutoring in the homes of the tutors or tutees; one-to-one, small groups, and large classes in a tutoring organization (Bray, 2013). Many studies have reported the positive impact of private tutoring on students’ academic performance in a variety of contexts (Mischo and Haag, 2002; Nath, 2008). Despite the prevalence, importance, and effectiveness of private tutoring, learners’ experience in and perceptions of English Private Tutoring (henceforth EPT), for study abroad preparation have been rarely researched. Therefore, this study investigates students’ experience in and perceptions of EPT for study abroad preparation. To pinpoint a more focused and comprehensive picture of such type of EPT in China, the study focuses on the teaching and learning of writing for study abroad tests in EPT, rather than examining students’ general experience of learning in that context.

## 2. Literature review

Private tutoring, a.k.a. shadow education, has been widely researched in a range of countries and regions^1^, e.g., in Bangladesh (Nath, 2008; Mahmud, 2021), Cambodia (Edwards et al., 2020), Canada (Aurini and Davies, 2004), China (Bray, 2013; Zhang et al., 2020), Egypt (Fergany, 1994), Hong Kong (Bray and Kwok, 2003; Yung, 2015, 2019, 2020), Ireland (Smyth, 2008), Germany (Mischo and Haag, 2002), Japan (Stevenson and Baker, 1992; Allen, 2016), South Korea (Lee, 2010; Exley, 2020), Turkey (Ünal et al., 2010), and Vietnam (Dang, 2007). Private tutoring in English is known as English private tutoring.

The existing studies on EPT have various foci such as the popularity and effectiveness of EPT, motivational orientations, tutors’ identity, and students’ reflections on EPT. As one of the earliest studies on this topic, Khuwaileh and Al-Shoumali (2001) explored the reasons and conditions for the prosperity of EPT among university students in Jordan. They found that the main reasons for the popularity of EPT included the lack of local textbooks, the importance of English, and the family’s economic condition. Hamid et al. (2009) examined the effect of EPT in Bangladesh and found that the students who took EPT were more likely to obtain a higher test grade. The positive effect of EPT was also found in Lee’s (2010) exploration of high school students in South Korea and Chou’s (2017) investigation into primary students in the Taiwan region. Different from these studies, Chang (2019) found that the time devoted to self-study activities had a more significant positive effect than that of EPT.

The motivational orientations in attending cram schools and students’ perceptions of EPT were another focus of EPT research. Chung (2013) found that instrumental and integrative motivation were two major factors for senior high school students to learn at cram schools. The students acknowledged the helpfulness of EPT for them to attain a higher exam grade, but not equally helpful in improving their communicative competence because the surface learning approach was the main learning approach adopted at cram schools. Similar results were reported in Yung’s (2015) investigation into Chinese learners’ reflections on EPT in Hong Kong, who had “ambivalent and paradoxical attitudes” toward EPT (Yung, 2015, p. 707). In a more recent study, Yung (2019) found varied motivations of students to engage in EPT, with one being students’ favor of the tutors in EPT than their school teachers.

Despite the plethora of research on EPT, they focused on EPT in general rather than a specific subject. In addition, to our knowledge, scarce research on EPT focused on that is exclusive for overseas test preparation, despite the large scale of such type of EPT. Therefore, this study researched students’ experience in EPT for study abroad test preparation with a focus on the tutoring of English writing. The following research questions guided the present study:

(1) What is Chinese students’ experience in writing in EPT?

(2) How do Chinese students perceive the effectiveness of EPT?

## 3. Methods

### 3.1. Research design, instruments, and procedure

This study, as a part of a larger research project, adopted a mixed-methods research design and triangulated data sources from a questionnaire and in-depth interviews. The questionnaire was developed based on the first author’s 3 years of teaching experience in a cram school in China’s Mainland and through interviews with Chinese students about their experience of learning English in cram schools. The questionnaire consists of three sections with the first part asking about participants’ general background information, the second part inquiring about students’ experience engaged in EPT, and the last part about students’ perceptions of the EPT. From those who agreed to participate in the interview, we selected 11 students to conduct in-depth interviews with.

### 3.2. Participants and data collection

We used convenience sampling and recruited Chinese students from the first author’s former colleagues who taught writing to international students in a large public university in the US. We recruited participants from this group of population because those students had the experience of preparing for overseas to have fulfilled the requirement of university admission.

An anonymous online questionnaire was distributed to a convenient sample of 256 Chinese students at a US university (187 valid responses were received) to elicit their experience and perceptions of receiving EPT in writing. The 187 participants’ ages ranged from 18 to 22, with an average of 18.7 years. Of the 187 participants, 117 were males and 70 were females; 160 were freshmen, 12 were sophomores, 13 were juniors, and two were seniors.

Most of the participants finished the questionnaire in approximately 10 min. At the end of the questionnaire, they were asked if they were willing to participate in follow-up interviews. Of those who agreed, 11 were chosen. Of the 11 interviewees, five were males and six were females. Except for one sophomore, all the others were freshmen. Table 1 is the interviewees’ profiles.

**Table 1:** Interviewees’ profiles.

Interviewee	Gender	Age	Year in school
Cathy	F	18	1
Wendy	F	18	1
Matt	M	19	1
Steven	M	19	1
Monica	F	18	1
Sarah	F	18	1
Jacob	M	18	1
Adam	M	18	1
Linda	F	20	2
Jasmine	F	19	1
Jack	M	19	1

All names used in the study are pseudonyms to ensure confidentiality. In the interview sessions, the participants were allowed to use either Chinese or English. All of them opted for Chinese, although some of them code-switched to English occasionally. All interviews (see the Appendix for the interview protocol) were audio-recorded and transcribed for analysis. The interview transcripts were shown to the interviewees to ensure the authenticity and reliability of the interview data.

### 3.3. Data analysis

The students’ responses to the closed-item questions and their demographic information were analyzed through mathematical calculation and categorization. Their responses to the open-ended questions were coded according to the content. For the interview data, the original Chinese transcripts were used to ensure no important information missing due to translation. The transcripts were translated only when they were used to present results. The interview transcripts were coded using the following coding scheme as shown in Table 2.

**Table 2:** The coding scheme.

Category	Subcategory	Topic	Code
1. Experience	a. Engagement in EPT of writing in cram schools		1-a
	b. Type	i. Large classes	1-b-i
		ii. Small group	1-b-ii
		iii. One-to-one tutoring	1-b-iii
	c. Time devoted to EPT in writing		1-c
	d. Reason	i. Test-taking strategies	1-d-i
		ii. More comprehensive instruction	1-d-ii
		iii. More detailed feedback	1-d-iii
		iv. Tailor-made instruction	1-d-iv
		v. Target the test genre	1-d-v
		vi. Influence of parents	1-d-vi
		vii. Influence of friends	1-d-vii
		viii. Other	1-d-viii
2. Teaching methods	a. Teaching test-taking strategies		2-a
	b. Teacher lecturing		2-b
	c. Using writing templates		2-c
	d. Writing in class		2-d
	e. Teaching patterns of organization		2-e
	f. Analyzing model essays		2-f
	g. Memorizing words and phrases		2-g
	h. Providing detailed feedback		2-h
	i. Other		2-i
Perceptions	a. Usefulness in test preparation		3-a
	b. Usefulness in improving writing skills	i. Organization	3-b-i
		ii. Logic	3-b-ii
		iii. Sentence structure	3-b-iii
		iv. Grammar	3-b-iv
		v. Genre	3-b-v
		vi. Vocabulary	3-b-vi
		vii. Nature of the course	3-b-vii
		viii. Other	3-b-viii
	c. Perception rationale	i. Tailor-made instruction	3-c-i
		ii. Personal writing models	3-c-ii
		iii. Ceiling effect	3-c-iii
		iv. Rigidness	3-c-iv
		v. Long-term planning	3-c-v

All the data were manually coded. The researchers developed the coding scheme based on the students’ responses to the questionnaire questions, the research questions of the present study, the interview questions, the researchers’ knowledge regarding English writing teaching in China, and teaching experience in cram schools. The coding scheme went through several rounds of changes and modifications in the process of coding. After the final version of the coding scheme was established, the researchers went through the data again to make sure they were coded consistently.

## 4. Results

This section presents the findings of the research questions and is divided into three subsections: students’ EPT experience, the teaching of writing in EPT, and students’ perceptions of EPT. It is worth reminding that the study focused on students’ writing in EPT. For brevity reasons, EPT in the following sections referred to EPT writing if not specifically explained.

### 4.1. EPT experience

#### 4.1.1. Choice of EPT

The majority (89.3%) of the students reported having received EPT for the writing sections of overseas tests in various ways, as shown in Table 3.

**Table 3:** The ways of EPT instruction students received.

Ways of EPT instruction	Number	Percentage
Small group EPT in cram schools	102	54.5%
One-to-one tutoring in cram schools	78	41.7%
Large classes in cram schools	60	32.1%
Home tutoring	11	5.9%
Did not receive EPT	20	10.7%

It can be seen that the most popular type was small group tutoring, i.e., a small group of students (about 5–10) took a class with one tutor in cram schools. One-to-one tutoring at the cram school had a high percentage, despite its high cost.

Students choosing different types of EPT can be attributed to three different reasons: financial status, writing proficiency, and personal preference. Financial status is an important factor. For example, for students whose family income is not high, large classes may match their needs. As Wendy said, “*one-to-one tutoring can cost up to $100 per hour. It’s totally beyond my parents’ budget*.”

In terms of writing proficiency, for those whose proficiency is low and thus cannot follow the pace of large classes, small classes or one-to-one tutoring may better meet their needs since, in those classes, teachers can tailor their teachings to individual students. In addition, students can also choose the type of classes based on their personal preferences. For example, some students prefer interacting with the teacher personally; others may prefer larger classes where they can interact with other students and avoid frequent individual interaction with teachers.

#### 4.1.2. Time devoted to EPT

The time the students spent learning writing in cram schools was considerable. The minimum time on EPT in writing in cram schools was 1 h every week, whereas the maximum was 20 h per week. On average the participants spent 4.5 h per week in cram schools for writing only. In addition, they devoted themselves extensively by receiving EPT in cram schools over a long period of time. The shortest range was 4 weeks, while the longest was 40 weeks. That is, concerning the total amount of time spent on EPT in writing, the shortest time was 2 h per week for 12 weeks totaling 24 h, and the longest was 6 h per week for 32 weeks totaling 192 h, with an average of 52 h. When the cost of EPT in cram schools was taken into consideration^2^, the students spent a substantial amount of money on EPT to pass the entrance requirement for undergraduate studies abroad. This finding indicated that cram schools had become the main source for learning English writing for most students choosing to study abroad, echoing other researchers who reported the popularity of cram schools in East Asia (e.g., Kwok, 2004; Chou, 2017). In the interview, Cathy reported that she even stopped going to school in her senior year. Instead, she spent 32 weeks learning English in a cram school and devoted 6 h to English writing per week. Students’ reliance on cram schools for overseas tests has “caused an exponential increase in the formal tutoring service business in China” (Zhang, 2019, p. 84)^3^.

To sum up, the students did not only spend a huge amount of time on EPT but a ton of money as well. Therefore, the reasons for the students’ devotion to and investment in EPT were also investigated.

#### 4.1.3. Reasons for engaging in EPT

The most reported reason for engaging in EPT was that the test-taking strategies taught in cram schools could help them obtain a higher score on overseas writing tests quickly, agreed by 71.5% of the 167 participants who engaged in EPT. Other popular reasons included the following: the writing teachers in cram schools provided comprehensive instruction in English writing (52.1%); the writing teachers in cram schools gave more detailed feedback (44.9%); the writing teachers in cram schools provided tailor-made instruction, which could help improve English writing quickly (37.1%); and high school teachers did not teach the type of writing in overseas tests (33.5%). Reasons that were not as popular included: parents wanted the students to; high school teachers did not teach English writing; friends went, so the students wanted to go with them; students could not understand what teachers taught in high school; and a lack of motivation to study on one’s own.

### 4.2. Teaching methods of EPT

The frequently employed teaching methods in EPT included (in decreasing order of frequency): teaching test-taking strategies (64.7%), lecturing (64.1%), providing writing templates (61.7%), asking students to write in class (58.1%), teaching different organization patterns (57.5%), analyzing model essays (56.9%), asking students to memorize words and phrases (53.9%), and giving detailed feedback (50.9%). Other less frequent activities included: asking students to imitate writing models, teaching grammar, practicing typing, asking students to read and imitate examples by famous writers, and asking students to write journals or diaries. It can be seen that teaching test-taking strategies was the main approach of cram schools, which was one of the bestselling points of cram schools. As reported earlier, over 71.5% of the participants received EPT writing in cram schools because examination-taking strategies taught in cram schools could help them obtain a higher grade on overseas writing tests quickly.

Since teaching test-taking strategies was the most reported teaching method, we asked students to further elaborate on the strategies in the interviews. Three common test-taking strategies were found: writing the introduction by using a fixed structure like “while some people believe… Others… From my perspective…,” elaborating short sentences into long and complex ones to demonstrate lexical and syntactic variety, and making full use of the reading passage in integrated writing. However, while such strategies are assumed to be useful for tests by cram school teachers, they may not help raise the test scores because the cliché pattern used in the introduction may not engage the raters, the too-long and complex sentences may be confusing and contain errors, and too much use of the expressions in the reading passage may lead to plagiarism. Such mismatch between teachers and raters in terms of what is good writing was previously reported by Qi (2007) who investigated high school teachers’ perceptions and test developers’ intentions.

Providing writing templates was also mentioned frequently in the interview, which echoes the result of the questionnaire. Students believed that the writing templates, especially those for integrated writing, were helpful in that using templates can save them time thinking about the language structure and expressions. Despite the acknowledgment of the usefulness of the writing templates, some students showed concerns and were afraid using writing templates might be accused of plagiarism, as Linda said,

*I heard ETS has a human rater and a machine rater for TOEFL writing. I’m afraid the machine rater can recognize the same expressions if too many people use the same writing templates. If accused of plagiarism, the consequence will be serious…. So I dare not use templates.*

### 4.3. Students’ perception of the effect of EPT

When it comes to the perceptions of the effect of EPT, the majority of the students (86.2%) believed EPT helped prepare them for writing in overseas tests like TOEFL and IELTS. To be more specific, students considered EPT useful in terms of helping them improve in organization, logic, sentence structure, vocabulary, and grammar, but rarely in the genre as shown in Table 4. This can be understood since the EPT was test-oriented and targeted the several genres that were tested in overseas tests.

**Table 4:** Perceptions of cram the usefulness of EPT.

	Useful		Useless	
	Number	Percentage	Number	percentage
Organization	89	53.3%	78	46.7%
Logic	87	52.1%	80	47.9%
Sentence Structure	85	50.9%	82	49.1%
Vocabulary	82	49.1%	85	50.9%
Grammar	80	47.9%	87	52.1%
Genre	39	23.4%	128	76.6%

The reasons for students’ perceptions of the effect of EPT can be found in the interviews. For example, Sarah thought EPT effective because of the tailor-made instruction, as illustrated in her interview excerpts:

*They offered me one-to-one tutoring. The teacher could focus on my problems and provide instruction that suits me, like tailor-made instruction specially designed for me. So, my writing improved quickly*.

Matt offered another reason:

*The cram school teachers taught us how to build our own writing models. Writing models were specific phrases or sentences that we could use to build logic and structure in writing. It saved a lot of time in TOEFL writing*.

The friendship between teachers and students in cram schools was also a positive influence, as indicated in Jack’s comment: “*My English teacher at the cram school spent more time with me, and we were just like friends. I was willing to accept more ideas and feedback from him*.”

Despite the acknowledgment of the usefulness of EPT in helping them prepare for tests, some students revealed their concerns, as Monica stated,

*“Although the writing instruction in cram schools could help me raise scores, it had a ceiling effect…. There is a limitation of the effect of the cram school instruction since it doesn’t radically improve my writing ability.”*

Other students complained that the teaching in cram schools was “*too rule-governed and modeled that they did not know how to write other genres except for TOEFL essays*” (Jasmine). It seems that for most students, the EPT instruction they received for test preparation was more useful in helping them meet the cut score requirements on the tests rather than improving their general writing abilities. Only a small portion of the students confirmed the role of EPT in improving their writing ability, as reflected in Cathy’s experience,

*Before I went to XXX (the cram school she attended), I couldn’t write more than 100 words and my vocabulary was no more than 3,000 words. But since I went there, my teacher had made a detailed plan for me… He asked me to memorize words, and practice using them in writing…. He taught me how to brainstorm ideas for writing, organize main points, and maintain good logic…. I spent six hours a week for 32 weeks learning writing at the cram school…. It takes time to feel the difference*.

As Cathy said, “it takes time to feel the difference.” For those who aim to raise scores in a short time, EPT tutors might have no time to make long-term plans to improve their writing ability slowly, but as Cathy spent the last year in the cram school preparing for overseas tests, the tutors could have more time to help her improve for the long-term goodness. It seems that the time spent on EPT can make a difference in teachers’ aims and ways of teaching.

## 5. Discussion

The present study found that Chinese students relied heavily on EPT for study abroad test preparation. Most of the students had engaged in EPT in various ways by devoting both intensively and extensively to EPT in cram schools, indicating that EPT for overseas test preparation was popular among Chinese students. The students’ substantial investment in EPT in cram schools reflected the long tradition of valuing education in China (Gao, 2008; Yung, 2015).

The teaching of the writing of EPT was mainly test-oriented considering their aim to help students obtain as high test scores as possible. The majority of students were positive about the crucial role of EPT in helping them prepare for writing tests; however, they were less confident about its effectiveness in improving their writing abilities in the long run. The different perceptions of usefulness indicate that the students held a complex attitude toward EPT, in line with the students’ mixed attitudes toward EPT reported by other scholars (Chung, 2013; Yung, 2015; Allen, 2016). These studies observed that students appreciated the meaningfulness of EPT in preparing them for exams, but not for authentic interaction and communication.

The reasons for such complex attitudes may lie in the manner of teaching in EPT. Cram school teachers spare no efforts to satisfy students’ needs because EPT is more of a service industry than an education industry, as a cram school administrator stated (Zhang, 2019). Hence, cram school teachers treat learners as not only their students, but also customers. To meet students’ needs, the teachers employ every means to help them achieve good grades, which correspond to the students’ major aims. Indeed, teaching test-taking strategies is also the bestselling point since most students engaged in EPT in cram schools for those *secret and magical* test-taking strategies. The methods can help students raise scores on writing tests and are deemed useful by most students. This finding is consistent with the positive impact of EPT in improving students’ test performance reported in other contexts, for example, in Bangladesh (Nath, 2008), Germany (Mischo and Haag, 2002), and Turkey (Ünal et al., 2010).

Concerning writing pedagogy, the most frequent activity was model-writing instruction. Such instruction may not have a long-term effect on students’ learning. Some students in the present study commented that they only used EPT for test preparation and admitted that they would not use it in their future study, similar to observations in Hong Kong students in Yung’s (2015) study. However, what is worth noticing is that different from Yung’s (2015) study, a small portion of the participants in the present study did believe that EPT at cram schools also helped them improve their language proficiency in the long run. Most of the students in the present study thought that the skills helped test preparation, whereas only a few deemed that some of the skills could be transferred to future learning, including vocabulary, grammar, and sentence structure. The latter usually spent a long time in EPT, which might explain the different perceptions among students. It is possible that the longer students devote to EPT and the more instruction they receive in EPT, the less of the *cramming* nature they are influenced by EPT. Given that the main goal of cram schools is to help students obtain the highest test score, the time students spend in cram schools can make a big difference in the way of teaching. For example, if students spend a short time in cram schools, the teachers must cram them for the test. However, if students devote a long time to EPT in cram schools, the teachers could set up a teaching plan that helps the students achieve a good test score by improving their general writing abilities step by step instead of only relying on test-taking strategies, thus weakening the nature of cramming caused by EPT.

Given the small sample size, interpretations of the data should be cautious. In addition, the generalizability of the findings is not the purpose of the present study and warrants a larger-scale study at the national level.

## 6. Conclusion

The present study investigated Chinese students’ experience in and perceptions of EPT for study abroad writing test preparation. The results showed that most students received EPT in writing in various ways at cram schools and they devoted themselves to EPT in cram schools intensively. They favored EPT in cram schools mainly because the test-taking strategies taught there could help them obtain a higher grade on the writing section in overseas tests. With respect to writing teaching in cram schools, the most frequent teaching activities included teaching test-taking strategies and providing writing templates. Although most students acknowledged the usefulness of EPT in writing in terms of preparing themselves for the writing test, it was not necessarily the case for improving their general writing abilities. The students believed that the writing instruction was test-oriented and had a ceiling effect in improving their general writing abilities. However, with enough long time in EPT, the cramming nature of cram schools can be less obvious.

The study has important implications for policymakers, applied linguists, TESOL practitioners, and university writing instructors. This study answers the call of scholars for more research to bridge private tutoring and language learning considering that EPT plays an important role in “shaping learners’ out-of-school L2 learning experiences” (Yung, 2019, p. 120). Since EPT has become an indispensable part of English learning for students, applied linguists and TESOL practitioners may need to consider their out-of-school EPT experience as well as their mainstream formal school education to form a complete picture of overseas students’ learning background. Additionally, the investigation of EPT in writing in cram schools can shed light on the field of second language writing as well by painting a panoramic picture of second language writing instruction and EPT, which is usually hidden from second language writing teachers and researchers.

## Data availability statement

The raw data supporting the conclusions of this article will be made available by the authors, without undue reservation.

## Ethics statement

Ethical review and approval was not required for the study on human participants in accordance with the local legislation and institutional requirements. Written informed consent from the (patients/participants OR patients/participants legal guardian/next of kin) was not required to participate in this study in accordance with the national legislation and the institutional requirements.

## Author contributions

CZ, YZ, and YG contributed to the conception of the study. CZ collected data and wrote the first draft of the manuscript. All authors contributed to the article and approved the submitted version.

## Funding

This study is based upon work supported by a grant awarded to the first author, CZ funded by the Shandong Provincial Teaching Reform (grant no. M2021186), and a grant awarded to the third author, YG funded by the Social Science Funding of Shandong Province (no. 19CWZJ27).

## Conflict of interest

The authors declare that the research was conducted in the absence of any commercial or financial relationships that could be construed as a potential conflict of interest.

## Publisher’s note

All claims expressed in this article are solely those of the authors and do not necessarily represent those of their affiliated organizations, or those of the publisher, the editors and the reviewers. Any product that may be evaluated in this article, or claim that may be made by its manufacturer, is not guaranteed or endorsed by the publisher.

## Footnotes

^1^For detailed review on shadow education in Asia and the pacific, see Bray (2022).

^2^The hourly cost of EPT per person in Mainland China ranges from $12 for large classes to $100 for one-on-one tutoring (Zhang, 2019).

^3^From July 24, 2021 on, China has launched a series of policies to tighten private tutoring, with most after-school private tutoring on academic subjects for K-9 students being restricted, but after-school education for non-academic subjects such as sports and art as well as EPT for overseas preparation is still allowed.

## Appendix: Interview protocol

1. Did you receive any private tutoring in cram schools?
2. What was your EPT experience?
3. What do you think of the EPT you received?
